# From exercise dose to social dose: a narrative review of the components, mechanisms, and design principles of group-based physical activity interventions to reduce loneliness

**DOI:** 10.3389/fpsyg.2026.1843858

**Published:** 2026-05-21

**Authors:** Lei Yu, Bo Zhou, Jiaxin Cai

**Affiliations:** 1 Hebei Normal University, Shijiazhuang, Hebei, China; 2 Hunan Institute of Science and Technology, Yueyang, China

**Keywords:** belongingness, group-based physical activity, intervention design, loneliness, narrative review, peer support, social dose, social identity

## Abstract

Loneliness is a growing public health concern associated with adverse mental, physical, and social outcomes. Group-based physical activity (GBPA) has emerged as a potentially scalable approach for reducing loneliness, but intervention effects are heterogeneous and are not adequately explained by exercise dose alone. In this narrative review, we propose the concept of *social dose* to complement conventional exercise dose. Whereas exercise dose refers to the frequency, intensity, and duration of activity, social dose refers to the structured exposure to opportunities for social connection embedded within group-based activity. We conducted a theory-guided narrative synthesis of literature from social psychology, exercise science, and public health, with emphasis on systematic reviews, intervention studies, qualitative studies, and foundational theoretical work relevant to loneliness, GBPA, belongingness, social identity, and peer support. We organize the evidence around three psychosocial pathways—belongingness, social identity, and peer support—and identify actionable intervention components including stable cohorts, repeated co-presence, predictable rituals, psychologically safe group climates, shared goals and symbols, buddy systems, peer mentoring, and inclusive leadership behaviors. We also discuss cross-cutting moderators at the individual, group, contextual, and activity-type levels, and outline implications for measurement and mechanism-sensitive trial design. We argue that GBPA should be understood not only as an exercise intervention, but also as a socially structured exposure intervention. However, the current evidence base remains heterogeneous, GBPA-specific causal evidence is uneven across pathways, and many studies underreport social-interaction components. Treating social dose as a measurable and designable construct may help make GBPA interventions more precise, equitable, and scalable for reducing loneliness.

## 1 Introduction

Loneliness has emerged as one of the major challenges in global public health. As a subjective experience of distress, loneliness refers to an individual’s perception of the discrepancy between the quality of their interpersonal relationship and their expectations (Baumeister and Leary, 2017). A growing body of evidence indicates that persistent loneliness not only undermines mental health but is also associated with cardiovascular disease, cognitive decline, and an increased risk of premature death (Holt-Lunstad et al., 2015). Against this background, there is an urgent need for accessible, scalable, and effective interventions that can reduce loneliness in everyday settings.

Physical activity interventions, particularly group-based physical activity (GBPA), have received increasing attention as a potential approach to reducing loneliness. Group activities such as exercise classes, community walking groups, ball sports, dance, and Tai Chi not only provide physical health benefits but also create natural opportunities for social interaction (Bennett et al., 2018). Previous reviews suggest that physical activity interventions may improve loneliness-related outcomes, yet substantial heterogeneity exists across studies, including marked variation in effect sizes (Shvedko et al., 2018). This heterogeneity reveals that not all group-based activities are equally effective in reducing loneliness—the key question is “what works, how it works, and under what conditions it works.” Importantly, loneliness is not entirely static; it can vary over time and across context, suggesting that the timing, predictability, and social quality of repeated group encounters may influence intervention effectiveness. This question is particularly relevant for aging populations, for whom social participation and meaningful community roles are central to successful aging. In this context, GBPA may help recreate structured opportunities for connection, visibility, and reciprocity in everyday life.

Traditional physical activity research has long focused on exercise dose, including frequency, intensity, and duration, because these parameters predict physiological adaptation. However, when the target outcome is loneliness, a subjective social experience, exercise alone is insufficient. The fact that interventions with comparable exercise doses can yield markedly different loneliness outcomes suggests that another dimension of intervention design is also important (Shvedko et al., 2018). This suggests that the effects of group activity on loneliness cannot be reduced to “how much one moves,” but should instead consider “how one moves together. Evidence from intervention-component analyses further suggests that social and interaction-related elements may be particularly important for loneliness outcomes (Ahn et al., 2024).

On this basis, this review introduces and examines the concept of “social dose.” Social dose refers to the structured exposure to opportunities for social connection embedded within a group intervention. It captures not only the quantity of interaction but also its quality and organization. In this review, social dose is operationalized across four key dimensions: (1) peer stability—whether participants repeatedly interact with the same group members; (2) interaction reciprocity—the availability of opportunities for two-way, responsive communication; (3) group meaning—whether shared objectives, team symbols, or rituals help transform the experience from “I attend a class” to “we are a group;” and (4) psychological safety—whether members perceive the group atmosphere as safe enough to permit interpersonal risk-taking, such as admitting inadequacy or seeking help (Edmondson, 1999). This definition supports viewing group physical activity as more than an exercise program.

## 2 Scope and approach

This narrative review uses social dose as a key concept for synthesizing the core components, underlying pathways, and actionable design principles through which GBPA interventions may reduce loneliness. This review does not attempt an exhaustive systematic search or quantitative synthesis because the questions addressed span interdisciplinary theory, diverse intervention formats, and multiple types of evidence. Instead, it adopts a theory-guided synthesis strategy: after defining the conceptual boundaries and broad inclusion logic, it integrates evidence and reasoning across three complementary pathways—belongingness, social identity, and peer support—and distills replicable and transferable intervention “active ingredients” to guide future study design and practice. Relevant literature was identified through targeted searches of PubMed, Web of Science, Scopus, and Google Scholar, together with backward citation tracking of key reviews and conceptual papers. Search terms combined loneliness-related concepts with GBPA formats and psychosocial mechanism terms such as belongingness, social identity, peer support, group cohesion, and psychological safety. We prioritized systematic reviews, intervention studies, qualitative studies, longitudinal studies, and foundational theoretical papers that were directly relevant to loneliness, group processes, or the social design of physical activity. Because this is a narrative review, the synthesis is interpretive and may be vulnerable to selection bias and uneven evidential strength across sections.

### 2.1 Literature scope and boundaries

This review focuses on GBPA formats in which participants engage in physical activity together in repeated, shared, or potentially interactive settings. Included formats comprise group fitness classes, community walking and running groups, team-based sports, dance and rhythm-based activities, and coordinated mind–body practices such as Tai Chi. Our focus is not on the physiological properties of these activities alone, but on their social architecture. Features such as group stability, opportunities for interaction, shared goals, norms, group climate, and leader behavior together constitute a designable social dose that may influence loneliness trajectories.

Regarding population scope, this review is not limited to a single age group, but explores evidence across three primary groups: adolescents, adults, and older adults. This stratification is not intended to compare which group shows “greater effectiveness,” but rather to recognize how differences in social needs, peer relationship patterns, participation motivations, and changes in accessibility constraints throughout life stages affect the accessibility and feasibility of a social dose. For instance, adolescents are more susceptible to peer assessment and exclusion; adult participation is generally constrained by time and role pressures; and older adults commonly experience limitations linked to functional capacity, transportation, and caregiving resources. These distinctions suggest that social-dose design should be tailored rather than imposed as a universal social structure across all groups.

Regarding outcome measures, this review treats loneliness as the primary outcome and emphasizes its subjective nature; that is, the key indicator is an individual’s appraisal of relationship quality and satisfaction with connection, rather than the quantity of social contacts or network size alone. To avoid conflating loneliness with social isolation, we treat social isolation as a related but distinct objective structural condition, while treating social support, belongingness, social identity, and experiences of connection as core proximal indicators and mechanistic clues. In other words, loneliness is the primary outcome to be explained, whereas social support, belongingness, and social identity are treated as proximal indicators or candidate mechanisms that help explain why, how, and under what conditions change occurs.

### 2.2 Narrative synthesis strategy

This review adopts a synthesis approach in which theory serves as the organizing framework and intervention components serve as the analytic language. The theoretical framework is derived from three pathways: the belongingness pathway emphasizes the role of acceptance, visibility, and psychological safety on loneliness; the social identity pathway stresses how a sense of “we-ness” offers a more stable source of connection via meaning and identity continuity; the peer support pathway focuses on how the presence and sufficiency of support shape experiences of loneliness and explain why someone might feel isolated even when surrounded by others. While distinct, these three pathways are also intertwined: belongingness relates more to “relational climate and safety,” social identity relates more to “shared meaning and joint self,” and peer support relates more to “reliable resources and responsiveness.” This structure allows the review to develop clear mechanistic explanations while accommodating evidence categories from various research traditions (experimental studies, longitudinal studies, qualitative analysis, and theoretical development).

Within this framework, the review identifies replicable intervention components as candidate “active ingredients.” Here, active ingredients do not imply that any single technique will inevitably produce effects; instead, they focus on design elements that are easily identifiable in different conceptualizations, can be implemented and monitored, and have a plausible link to the three pathways. For example, fixed groups and stable attendance patterns can be regarded as structural components that increase “repeated co-presence and familiarity;” newcomer onboarding scripts, low-threat interaction rituals, and inclusive norms can be regarded as process-oriented components that foster belongingness; team symbols, shared goals, and role differentiation can be regarded as symbolic components that improve social identity; and paired support, structured feedback, and leader behaviors that facilitate connection can be regarded as supportive components that increase peer support quality. Through this mapping of components, mechanisms, and outcomes, we aim not only to identify intervention forms that may work but to clarify how interventions may be designed for greater effectiveness, offering a structured and actionable conceptualization for subsequent measurement, comparative design, and implementation evaluation. Components were retained as active ingredients when they recurred across intervention contexts, theoretically central to one or more pathways, or supported by qualitative or implementation evidence suggesting practical importance.

## 3 Findings

### 3.1 Overall logic chain: from group structure to psychosocial pathways and loneliness-related outcomes

**Figure 1:**
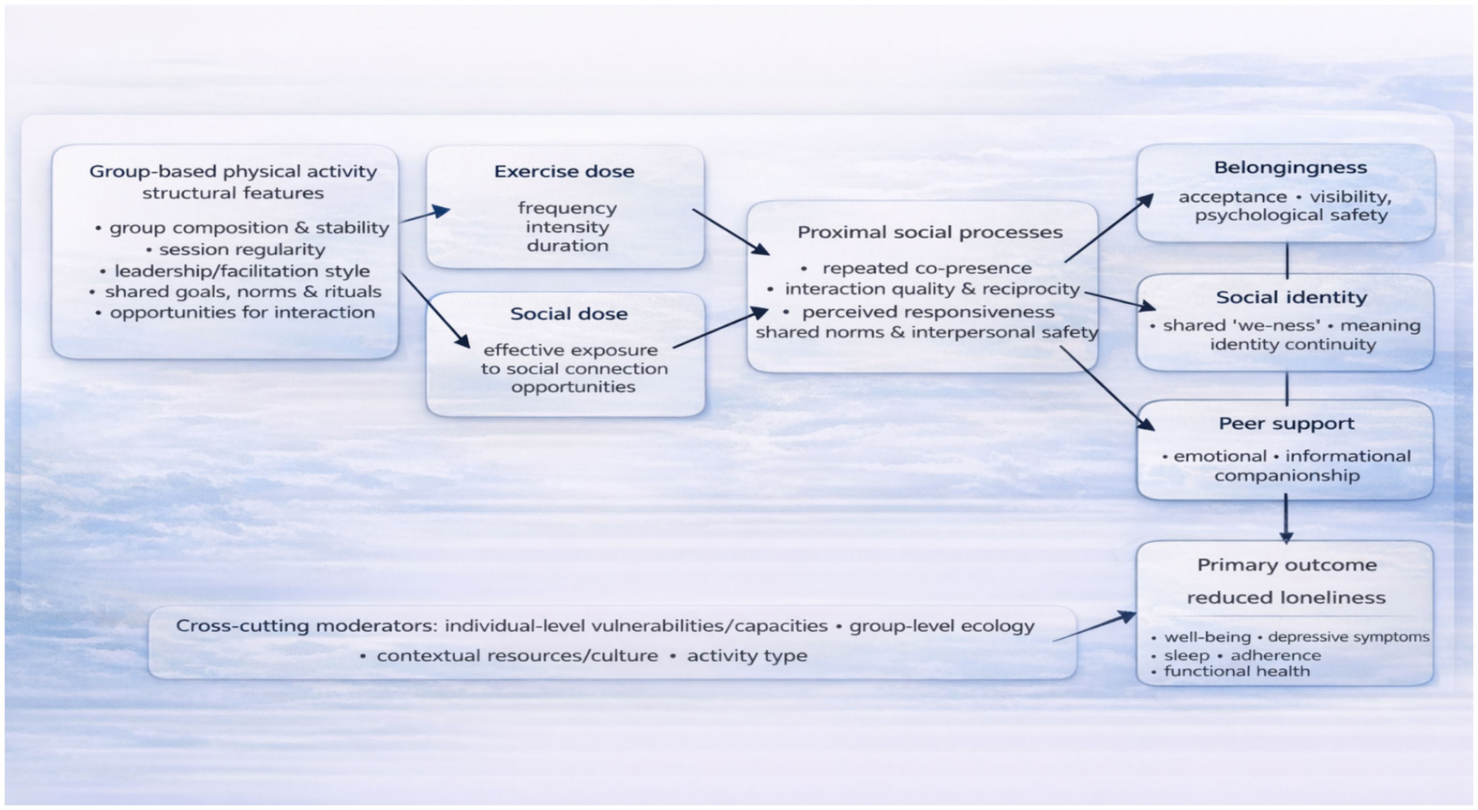
Conceptual framework linking group-based physical activity, social dose, psychosocial pathways, and loneliness reduction.

GBPA interventions can be understood as multifaceted social-exposure interventions rather than as exercise-only programs (Figure 1). We propose an overarching logic chain through which structural features of the group exercise setting (e.g., the composition and stability of the group, the consistency of sessions, the style of leadership and facilitation, common objectives and norms, and chances for interaction). Shape proximal social pathways— especially (i) frequency of interactions and consistent co-presence, (ii) relationship quality and perceived responsiveness, and (iii) shared norms and a sense of interpersonal security. These proximal pathways, in turn, activate three partly overlapping psychosocial pathways—belongingness, social identity, and peer support—that plausibly reduce loneliness and promote downstream health-associated outcomes.

This framework aligns with broader public health perspectives that recognize social connection as a determinant of health and with evidence that deficits in social connection, including loneliness and social isolation, are associated with increased morbidity and mortality risk (Holt-Lunstad et al., 2015). It is also consistent with evidence suggesting that physical activity interventions, including group-based formats, may influence loneliness-related outcomes. However, the observed effects are heterogeneous and likely depend on intervention design and contextual features (Shvedko et al., 2018). GBPA may also improve well-being, depressive symptoms, sleep, and adherence to activity, and possibly cardiometabolic or functional outcomes, through sustained participation, particularly in older adult trials and feasibility studies (Shvedko et al., 2020). More importantly, this framework suggests that reductions in loneliness are not an automatic consequence of increased exercise dose. Instead, changes in loneliness depend on the quality and structure of social interactions—referred to as the ‘social dose’—and on factors at the individual, group, and environmental levels.

### 3.2 Exercise dose vs. social dose: a complementary dosing model

Traditional exercise prescription emphasizes exercise dose—frequency, intensity, and duration—because these parameters predict physiological adaptation. However, when the target outcome is loneliness, an equally important dimension is social dose, that is, the structure and quality of social exposure embedded within the intervention.

#### 3.2.1 Exercise dose

Exercise dose is how much and how hard participants move: session frequency (e.g., weekly), duration (minutes), and intensity (light–moderate–vigorous). Exercise dose remains important because physical activity can influence mood, sleep, and self-regulation, which may, in turn, indirectly affect social functioning and willingness to engage socially. Nonetheless, dose-equivalent interventions can generate divergent loneliness outcomes, suggesting that exercise dose is insufficient to explain effects on loneliness (Shvedko et al., 2018).

#### 3.2.2 Social dose

We define social dose as the structured exposure to opportunities for social connection offered by the group format. In this review, social dose is operationalized across four dimensions: peer stability, or how often participants encounter the same individuals; interaction reciprocity, or opportunities for reciprocal and meaningful interaction rather than simple co-presence; group meaning, or shared goals, symbols, and rituals that help shift the experience from “I attend a class” to “we are a group;” and psychological safety, or a shared understanding that the group is safe for interpersonal risk-taking, such as admitting low fitness or seeking help. Psychological safety is an established construct in group and team research and is shaped by leadership and contextual support (Edmondson, 1999).

This social-dose lens also helps integrate findings from intervention-component research showing that ingredients linked to social interaction and group processes are particularly relevant when loneliness is the target outcome (Ahn et al., 2024).

### 3.3 Pathway 1: belongingness

#### 3.3.1 Theoretical grounding

Belongingness theory proposes that humans have a fundamental motivation to form and sustain lasting, positive interpersonal relationships; when this need is unmet, loneliness is more likely to emerge and persist (Baumeister and Leary, 2017). From this perspective, loneliness is not simply a matter of being alone; rather, it reflects a perceived gap between desired and actual relational connections. Therefore, it should be sensitive to conceptualizations that reliably offer acceptance, inclusion, and relational responsiveness.

#### 3.3.2 How group-based PA can build belonging

##### 3.3.2.1 Repeated exposure and familiarity

Regular contact with the same individuals can decrease uncertainty and increase positive evaluations over time, even before deep interaction occurs. The mere exposure effect provides a useful psychological principle: repeated exposure to a stimulus, including other people, tends to increase positive affect toward it, particularly when the initial valence is neutral or not strongly negative (Zajonc, 1968). In GBPA, this implies that regular sessions and stable groups can gradually transform strangers sharing the same space into familiar others, thereby reducing social anxiety and facilitating connection.

##### 3.3.2.2 Rituals and predictability

Belonging is fostered when a social setting is predictable: fixed time/place, consistent session scripts (opening/closing routines), and recurring milestones. Predictability reduces cognitive load and social ambiguity, which is particularly relevant for individuals high in social anxiety or who have experienced prior exclusion. This design principle is frequently reflected in qualitative accounts of participation in group-based PA, where a regular structure and clear expectations promote comfort and ongoing participation (Bennett et al., 2018).

##### 3.3.2.3 Psychological safety and non-judgmental norms

For belongingness to develop, participants should perceive the group as safe for interpersonal risk—e.g., disclosing low fitness, making mistakes, or asking for alterations. Psychological safety is understood as a shared belief that the setting is safe for interpersonal risk-taking and is shaped by leadership behavior and contextual support (Edmondson, 1999). In GBPA, instructors who model inclusive language, normalize various ability levels, and effectively avoid mocking/competition-overheating can increase the likelihood that repeated co-presence becomes genuine inclusion.

#### 3.3.3 Actionable components that map onto belongingness

From a belongingness perspective, intervention components are actionable when they foster stable, low-threat, and repeated opportunities for inclusive contact: stable cohorts and small-group subdivision through closed groups, fixed small teams, or consistent “pods” may increase familiarity and reduce the social uncertainty that hinders connection; greeting and check-in rituals, together with micro-acknowledgements such as structured name use, brief check-ins, and consistent opening greetings, build low-cost experiences of being seen that function as proximal signals of inclusion and accumulate into sustained belonging across repeated sessions, especially for individuals at risk of loneliness; shared milestones and joint celebrations through group-based progress tracking may strengthen inclusion and group continuity when these activities are framed to minimize harmful social comparison; and newcomer onboarding and buddy systems may reduce the common pattern in which newcomers attend once but fail to connect socially by lowering ambiguity and interpersonal threat early in participation. Evidence syntheses focused on components related to physical activity and loneliness further emphasize that “social- and interaction-related ingredients” are commonly critical for reducing loneliness, even across highly diverse intervention formats (Ahn et al., 2024).

#### 3.3.4 Evidence map: what kinds of studies support “belongingness→loneliness” in GBPA?

A systematic review and meta-analysis of randomized controlled trials in older adults assessed physical activity interventions targeting social isolation, loneliness, or low social support. They provided quantitative evidence that such interventions, including group-based formats, may affect these outcomes, albeit with heterogeneity across intervention categories and measures (Shvedko et al., 2018). Importantly, this heterogeneity indicates that simply “adding exercise” is insufficient; interventions with stronger social components may be more likely to show stronger loneliness-related effects.

PAIL (Physical Activity Intervention for Loneliness) is a randomized feasibility study in community-dwelling older adults at risk of loneliness, demonstrating the feasibility and acceptability of explicitly loneliness-oriented PA programming and providing practical insight into retention and delivery challenges (Shvedko et al., 2020). Feasibility work is particularly valuable for belongingness because it generally provides process data (attendance, acceptability, qualitative feedback) that helps identify “where belonging fails or succeeds.”

Qualitative analyses of GBPA trials, such as the GOAL trial, offer rich accounts of how older adults experience group programs. Such evidence generally identifies themes consistent with belongingness processes, including comfort derived from familiar faces, enjoyment of inclusive atmospheres, and the importance of program structure for feeling part of something (Bennett et al., 2018). Although qualitative data do not establish causality, they strengthen the plausibility of the process and guide design principles. Collectively, this evidence indicates that belongingness is a plausible pathway and that its effects are likely to depend in part on how effectively the program transforms repeated co-presence into a sense of inclusion.

#### 3.3.5 Boundary conditions and risks: when belongingness fails (or backfires)

A belongingness-oriented framework also predicts several failure modes, including exclusion dynamics and subtle gatekeeping, social comparison and ability-based shame, and mismatches between session structure and participant vulnerability. Open, drop-in formats may also have low continuity because irregular attendance and frequent changes in membership prevent the accumulation of familiarity and thereby hinder the development of belonging (Edmondson, 1999; Zajonc, 1968). A key design implication is that mitigating these risks requires the deliberate incorporation of social dose into intervention design, including stable micro-groups, explicit inclusion norms, instructor-facilitated introductions, and reduced evaluative social comparison, especially for groups already experiencing high levels of loneliness.

### 3.4 Pathway 2: social identity

#### 3.4.1 Theoretical grounding

The social identity approach is based on the idea that individuals can see themselves not only as distinct persons (“I” or “me”) but also as members of psychologically meaningful groups (“we” or “us”). Social identity theory (SIT) proposes that group memberships become part of the self-concept and acquire emotional and evaluative significance, shaping perceptions, motivations, and behavior in approaches that differ from purely interpersonal mechanisms (Tajfel and Turner, 2004). Self-categorization processes help explain when and how a shared identity becomes salient. Identity formation refers to the gradual development of meaningful group membership over time, whereas identity salience refers to the situational activation of that membership in a given context. In certain situations, people shift from a personal identity to group-based self-definition, adopting group norms and typical behaviors (“what people like us do”). This helps explain why group settings can generate powerful social influence and why the same activity, such as exercise, may have different psychosocial consequences depending on whether it is experienced as a personal routine or as participation in “our group.” (Tajfel and Turner, 2004). The ‘social cure’ approach views shared social identity as a major mechanism by which being part of a group benefits health—because it can furnish meaning, purpose, belonging, support, and joint efficacy—and thereby buffer against risks associated with social disconnection (Haslam et al., 2022). In physical-activity conceptualizations specifically, reviews of the social identity approach argue that internalizing group membership can shape engagement, group dynamics, and leadership, offering a theoretically grounded account of why group-based formats may yield benefits beyond exercise dose (Stevens et al., 2017).

#### 3.4.2 Identity formation in group exercise

GBPA can function as an identity-building context in which repeated participation gradually transforms a set of people exercising near each other into a psychologically meaningful in-group. This transformation is not automatic, but it is more likely when the setting provides recognizable group boundaries, shared meanings, and opportunities to enact a collective “we” through shared routines, norms, and narratives. The social identity approach proposes that once participants perceive the group as self-relevant (“this is my group”), the group may become a more durable source of connection—often more robust than transient social contact—because it is anchored in the self-concept (Haslam et al., 2022). Mechanistically, GBPA fosters such group identity via four core channels: shared goals and joint striving (e. g., “we are training for X”), where the pursuit of common objectives strengthens perceptions of shared fate and mutual relevance, making the group psychologically meaningful (Tajfel and Turner, 2004); shared language and symbols like nicknames, jargon, and team colors, which serve as identity markers that distinguish “us” from “not-us,” increasing category salience and ensuring continuity across sessions (Tajfel and Turner, 2004); developing norms that set expectations for newcomer treatment and define success helps turn a class into a cohesive community, influencing behavior even without explicit guidelines (Tajfel and Turner, 2004); and identity-relevant leadership, whereby leaders actively cultivate a sense of shared “we-ness,” establish inclusive norms, and acknowledge a range of contributions. Analyses in sport, exercise, and health contexts suggest that leadership is an important leverage point for identity-based effects (Stevens et al., 2017). Empirically, evidence from social settings demonstrates that the well-being benefits of physical activity are particularly pronounced when participation is internalized as a meaningful aspect of the self, which aligns with the identity pathway’s core emphasis on self-relevance instead of the mere co-presence of individuals (Inoue et al., 2024).

#### 3.4.3 Core components that scaffold social identity

From an intervention-design perspective, the identity pathway implies that GBPA should be designed not only to deliver movement but also to support identity formation through deliberate actions that make the collective “we” visible in an inclusive way. This can be operationalized via four core design strategies: (a) Team naming and symbols, where a group name, simple branding like a logo or color, or common items such as badges and stickers or T-shirts can enhance group distinctiveness and continuity, with the practical caveat that all symbols should signal inclusion (e. g., “any level welcome”) instead of exclusivity; (b) Shared goals, events, and joint milestones, where group challenges like joint distance targets, shared events like fun runs or local tournaments, and joint milestone rituals comprising celebrations for a “first month completed” transform individual progress into joint meaning, shifting motivation away from the individual “I should exercise” to the communal “we do this together”—a hallmark of identity-based engagement (Stevens et al., 2017); (c) Role differentiation and contribution diversity involve legitimizing and assigning diverse roles, which can enhance members’ perceived value to the group and strengthen investment and retention; (d) Online platforms for maintaining identity continuity (with distinct boundaries), where group conversations occur and online communities can sustain group identity between face-to-face meetings by fostering a sense of continued belonging, enhancing perceived continuity, and facilitating easy social connections. Emerging evidence on on-demand and online group exercise platforms suggests that social identification mechanisms may shape participation even among partly anonymous members, while also highlighting design tensions involving agency, anonymity, and community interaction (Richards et al., 2026), with an essential guideline that these online extensions should focus on promoting inclusion and coordination while minimizing harmful social comparison, harassment, and excessive pressure to participate.

#### 3.4.4 Evidence and testable predictions

##### 3.4.4.1 Evidence that identification links to loneliness reduction

A well-documented example comes from community evidence applying the social identity approach: across three studies, community identification predicted lower loneliness and higher well-being, with mediation patterns involving perceived support and loneliness (McNamara et al., 2021). Although not specific to exercise, it illustrates the core premise of this pathway: identification may help reduce loneliness. More directly, social-identity–derived interventions provide stronger causal support. Groups 4 Health (G4H), designed to build and sustain social group memberships, showed superiority over treatment-as-usual for reducing loneliness and social anxiety in a randomized controlled trial (Journal of Consulting and Clinical Psychology; DOI reported in repository records), supporting the plausibility of identity mechanisms as “active ingredients” for loneliness reduction (Haslam et al., 2019). A complementary review that integrates identity-based connections suggests that interventions fostering effective social identities may help reduce loneliness and mental health issues, encouraging identity-focused design within GBPA (Haslam et al., 2022).

##### 3.4.4.2 Testable predictions

The temporal-order hypothesis suggests that increases in exercise-group identification should precede decreases in loneliness rather than merely co-varying with them (Häusser et al., 2020). Likewise, the effects of social-dose elements, such as stable groups and shared symbols, are likely to be partly mediated by group identification, with further downstream effects through perceived belonging and social support (Häusser et al., 2020). Regarding dose–response relationships, stronger and more positive group identification—especially self-investment dimensions such as solidarity and group satisfaction—should predict greater decreases in loneliness (Leach et al., 2008). Moreover, leadership may serve as an important pathway-shaping factor, because identity-aligned leadership can strengthen the link between group identification and loneliness reduction by promoting inclusive norms and shared meaning (Stevens et al., 2017).

##### 3.4.4.3 Assessment recommendations (identity+engagement)

Social identification measures should include a multicomponent assessment of group identification, distinguishing between self-definition and self-investment to clarify which specific dimensions of identity (like unity or centrality) are most strongly predictive of loneliness reduction (Leach et al., 2008); for studies that involve longitudinal or repeated assessments and need to be concise, the validated Single-Item Social Identification (SISI) scale provides a reliable and practical option (Postmes et al., 2013). Participation density and engagement can be operationalized using metrics comprising attendance regularity, the proportion of stable group members, and online interaction indicators like message reading/posting rates and the ratio of supportive to comparative content, which measures ‘identity maintenance’ throughout sessions and between in-person meetings (Richards et al., 2026).

#### 3.4.5 Heterogeneity and potential adverse effects

The same identity mechanisms that underpin the “social cure” can also introduce important risks, particularly when group identity becomes exclusive, highly evaluative, or threatened. Exclusion and out-grouping may emerge because social identity theory emphasizes that individuals often aim for their in-group to stand out effectively; if a culture of GBPA implicitly values superiority, it may promote gatekeeping (e.g., labeling “real exercisers” or “real runners”) and exclude newcomers or those with lower fitness, which runs counter to the goals of reducing loneliness (Tajfel and Turner, 2004). Excessive competition and ‘identity pressure’ can occur when strong group identification heightens the pressure to conform to norms, such as training via injury or avoiding “letting the group down.” According to social identity research, a common identity can sometimes intensify stress reactions and social-evaluative threat, representing a potential “social curse” mechanism that requires tracking in performance-centered environments (Schury et al., 2020). Identity threats and transitions increase vulnerability: when group membership is key to one’s self-concept, disruptions such as injury, moving, or leaving can create identity discontinuity and psychological risk; recent analyses of sports contexts highlight the mental health consequences of unstable social identities during transitions, a concern that is particularly pertinent to exercise groups, especially among individuals with limited social resources (Stevens et al., 2024). A core design implication is that identity scaffolding in GBPA should be inclusive and flexible, providing multiple pathways to belonging, appreciating various roles and types of contributions, with clear norms against shaming and a structured “return/rehabilitation pathway” that preserves group membership during periods of disruption.

### 3.5 Pathway 3: peer support

#### 3.5.1 Theoretical grounding

Within social support research, support is generally understood as a multidimensional construct that can be measured as received support (particular actions that provide support) or perceived support (the belief that adequate support is available), with distinct implications for loneliness and health outcomes (Gottlieb and Bergen, 2010). In the loneliness literature, support functions closely aligned with emotional closeness (attachment) and social integration are particularly relevant: deficits in close attachment and social integration are theorized to underlie distinct forms of loneliness (emotional vs. social loneliness), and research grounded in Weiss’s framework of social provisions has uncovered distinct links between specific social provisions and various loneliness dimensions (Russell et al., 1984). For GBPA interventions, it is meaningful to differentiate four widely recognized support functions: emotional support (empathy, validation, warmth; conveying “you matter to us”), instrumental support (providing practical help to reduce obstacles like transportation, reminders, or modifications to exercise), informational support (guidance and expertise to enhance coping skills and proficiency, including technique instruction, pacing methods, and injury avoidance), and companionship support (engaging in activities together, ‘not doing it solo,’ and participating in leisure activities as a group). A critical insight for loneliness-centered GBPA frameworks is that loneliness may be more strongly linked to the perceived availability, fit, and adequacy of support than to the quantity of supportive acts alone. In a large meta-analysis of 177 studies involving more than 113,000 participants, social support was moderately associated with lower loneliness (r ≈ −0.39), with perceived support showing a stronger association than other forms, and support from friends being particularly salient (Zhang and Dong, 2022). This pattern suggests that peer-based support components, instead of instructor-only delivery, may be vital for targeting loneliness in GBPA interventions.

#### 3.5.2 Peer-support mechanisms in group-based physical activity

We conceptualize the peer-support pathway as a set of social mechanisms via which GBPA reduces loneliness by making participants feel supported in ways that (a) reduce obstacles to participation, (b) build skills and self-confidence, and (c) create reliable experiences of “someone is with me.”

##### 3.5.2.1 Barrier reduction + “showing up is easier with someone”

Peer support and companionship can help reduce practical and psychological barriers such as uncertainty, intimidation, transport difficulties, and fear of being judged. Process evidence from peer-support physical activity delivery for older adults suggests that high social engagement fostered through peer-volunteer interaction may increase adherence, a vital precondition for repeated exposure to supportive relationships (Crozier et al., 2020).

##### 3.5.2.2 Self-efficacy building via mastery experiences and social persuasion

Social Cognitive Theory argues that self-efficacy is strengthened via mastery experiences, vicarious learning, verbal persuasion, and physiological and affective states (Bandura, 1977). Peer encouragement and shared success in GBPA may reflect these sources of self-efficacy, as peers can normalize struggle, model coping, and offer credible verbal encouragement (“you can do this”), thereby increasing both competence and willingness to engage socially. Recent PA evidence continues to frame social support as an environmental resource that can build exercise self-efficacy (directly mentioning mastery experiences and verbal encouragement), reinforcing the plausibility of this mechanism in group exercise conceptualizations (Wu et al., 2026).

##### 3.5.2.3 From “support acts” to “support availability” (perceived support)

Loneliness is strongly shaped by the perception that support is available and trustworthy. In a study distinguishing perceived vs. received support, perceived integration (belonging) and attachment (emotional closeness) emerged as strong predictors of lower loneliness, even after controlling for overall received support—consistent with the idea that loneliness may change when support becomes psychologically dependable instead of merely present (Lau et al., 2025).

#### 3.5.3 Core components (“active ingredients”) for operationalizing peer support in GBPA

Designing peer support into GBPA interventions is not simply a matter of increasing social interaction, but of structuring reliable and need-responsive support exchanges through intentional design strategies: (a) Buddy pairing and small-group mutual help, which involves pairing newcomers with a peer buddy for the first 2–4 sessions (only after rotations should occur only after social comfort has been established) and allocating straightforward collaborative micro-tasks (e.g., tracking each other’s workout speed and exchanging a brief personal ‘win and challenge’ after the session); this approach boosts companionship support, reduces entry-level social anxiety, and fosters stable support dyads that lay the groundwork for perceived support availability (Zhang and Dong, 2022). (b) Peer mentors and peer volunteers may act as support catalysts, particularly in community and older-adult programs, by initiating conversations, noticing absence, and promoting inclusion, frequently with a less noticeable social hierarchy compared to professional staff; delivery evidence on peer-supported physical activity interventions highlights social engagement and peer relatability as core pragmatic facilitators, while also noting critical implementation requirements, including clear role definition, targeted training, and coordination with qualified professional staff (Crozier et al., 2020). (c) Coaches or leaders act as facilitators of connection by executing four main actions: actively introducing group members, normalizing varying ability levels, and modeling supportive group norms (e.g., “we help each other”), and actively handling adverse social interactions. This extends beyond an optional feature and instead represents a fundamental part of the social dose that enhances the quality of support, and evidence on psychological safety shows that leader behaviors and group norms shape whether participants feel safe taking interpersonal risks (e.g., admitting exercise difficulty)—a prerequisite for seeking and receiving support (Knopes and Dégale-Flanagan, 2025). (d) Structured interaction routines, such as short, predictable pre-session check-ins and post-session reflections that transform parallel participation into meaningful support exchange without requiring advanced social skills, alongside structured feedback practices that prioritize encouragement and problem-solving over evaluative judgment; this design enhances informational and emotional support while shielding socially vulnerable participants from the uncertainty of unstructured social interactions. (e) Online channels for support continuity, framed around “low-intensity support” instead of surveillance, where peer online communities sustain companionship and send gentle participation reminders between in-person sessions, with explicit privacy norms and clear boundaries in place (see section 3.5.5); evidence from digitally enabled peer support interventions demonstrates clinically meaningful reductions in loneliness in real-world cohorts, supporting the broader feasibility of peer support as an anti-loneliness mechanism—even as GBPA-specific digital hybrid models still require targeted empirical assessment (Bravata et al., 2023).

#### 3.5.4 Why “support quality” matters more than “support quantity”

A persistent puzzle in evidence on loneliness is why some individuals can be “in groups” yet remain lonely. The peer-support pathway offers a clear explanation: loneliness is influenced by whether support is seen as appropriate, responsive, and dependable, rather than by the mere presence of others or how often interactions occur.

##### 3.5.4.1 Perceived support appears more strongly associated with loneliness than received support

The meta-analysis published in Acta Psychologica indicates that perceived social support shows a stronger negative association with loneliness than other forms of support, and friend/peer support tends to be particularly important (Zhang and Dong, 2022). This aligns with evidence that perceived integration and attachment predict lower loneliness above and beyond general received support, suggesting that loneliness decreases when people internalize the belief that support is available rather than simply counting supportive acts (Lau et al., 2025).

##### 3.5.4.2 Support adequacy/matching: the “platinum rule” problem

Support may fail (or backfire) when it is mismatched to the recipient’s needs—e.g., giving advice when validation is needed, pushing intensity when safety is needed, or “cheering” in approaches perceived as patronizing. Support-adequacy frameworks explicitly define effective support as a match between desired and received support, arguing that adequacy is a core determinant of relational outcomes beyond amount (Brock and Lawrence, 2010). Optimal-matching perspectives emphasize the importance of aligning the type of support with the specific stressor or need; in loneliness-focused GBPA, this means designing interactions that foster warmth, inclusion, and social integration rather than providing advice or logistics alone (Cutrona, 1990).

##### 3.5.4.3 “Superficial sociability” vs. “responsive support”

Individuals may experience high interaction frequency but low responsiveness (e.g., small talk without recognition; presence without care), resulting in “lonely in a crowd.” “This is the reason GBPA designs that only boost co-presence, like large drop-in classes, may not perform as well as those that foster mutual recognition, such as small stable groups, buddy systems, and leader-promoted introductions. The suggestion is that social dose should be defined by the quality and consistency of interactions, rather than merely the duration of social exposure (Zhang and Dong, 2022).

#### 3.5.5 Risks and ethics: dependency, boundary issues, privacy, and safety

**Figure 2:**
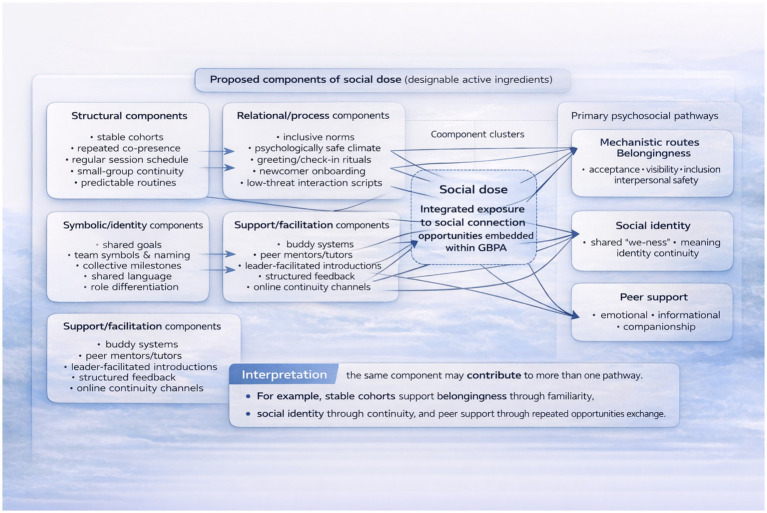
Proposed components of social dose and their mapping onto belongingness, social identity, and peer-support pathways in group-based physical activity interventions.

Embedding peer support within GBPA interventions introduces ethical considerations that are commonly underdefined in exercise program design, with four core areas requiring intentional attention: (a) Over-dependence and boundary ambiguity, as peer support relationships can shift toward over-involvement—such as peers feeling undue responsibility for another’s wellbeing or participants expecting quasi-clinical levels of availability from peers; research on peer support ethics highlights that the boundaries in these connections are typically flexible and context-specific, necessitating deliberate design and explicit discussion instead of assuming that informal interactions are inherently harmless (Knopes and Dégale-Flanagan, 2025). (b) Confidentiality and sensitive disclosures, given that loneliness-targeted GBPA programs may prompt participants to disclose personal experiences related to mental health, trauma, or suicidality; peer-support guidance materials recognize confidentiality as a cornerstone of trust, while also outlining important exceptions for situations involving safety risks (e.g., stated intent to harm oneself or others), which for GBPA means establishing a clear escalation pathway to trained professional staff or specialized services and setting transparent expectations for participants about what peer supporters can and cannot reasonably keep confidential. (c) Safety, safeguarding, and power dynamics, particularly for programs serving vulnerable populations like older adults living alone, adolescents, or those with chronic illness, which should pre-specify robust safeguarding practices, including clear role definition for peer mentors, basic training in supportive communication, and explicit group norms against harassment or coercive pressure to participate; evidence from implementing peer-supported physical activity programs further emphasizes practical challenges—such as consistent eligibility mechanisms for peer mentors, standardized induction training, and environmental risk assessment—that directly intersect with ethical delivery and participant safety (Crozier et al., 2020). (d) Online extensions: privacy and norm governance, as digital peer support channels like group chats require explicit, agreed-upon privacy norms—including no unsanctioned sharing of others’ personal information, informed consent for photo sharing, and an expectation of respectful communication, along with structured moderation strategies to handle interpersonal disputes and unwanted social comparisons, and other harmful online interactions that could compromise the program’s ethical and supportive goals. Figure 2 shows the proposed components of social dose and their mapping onto belongingness, social identity, and peer-support pathways in GBPA interventions.

### 3.6 Cross-cutting moderators

Across the three pathways—belongingness, social identity, and peer support—the effects of GBPA on loneliness are unlikely to be uniform. Instead, outcomes are shaped by who participates, how the group is structured and led, and the broader context in which the program is embedded, including infrastructure, culture, and digital conditions. The same social-dose feature, such as stable groups, may therefore act as a facilitator for some participants but as a barrier for others.

#### 3.6.1 Individual-level moderators

Personality, particularly extraversion and neuroticism, is a core moderating factor, as meta-analytic evidence shows that extraversion is negatively associated with loneliness, neuroticism is positively associated with loneliness, and other personality traits show weaker relationships (Buecker et al., 2020). This has direct implications for GBPA, as extraversion may lower the threshold for initiating social interaction and thus increase the actual social dose experienced, while neuroticism can heighten perceived social threat and rejection sensitivity—undermining feelings of belongingness and perceived support even when objective social contact is present. Individual personality features may influence both the initial uptake of GBPA and the long-term maintenance of its social benefits (Tavernier et al., 2016). Social anxiety represents another critical moderator: it decreases participants’ willingness to take part in group interaction. It amplifies the negative interpretation of ambiguous social cues, thereby weakening all three core pathways of GBPA’s anti-loneliness effects—especially the belongingness pathway (driven by fear of social assessment) and the peer support pathway (stemming from difficulty seeking help). A population-based cross-lagged panel analysis spanning multiple years documents reciprocal associations between loneliness and social anxiety symptoms in the general population, pointing to potential feedback loops where loneliness exacerbates social anxiety and social anxiety in turn deepens loneliness (Reinwarth et al., 2024). For GBPA design, this means that unstructured social time may not benefit socially anxious participants and may even burden them; instead, structured, low-risk interaction scripts, such as short regular check-ins combined with introductory activities, and explicit non-judgmental group norms, become vital social safety scaffolds. Depression (and affective burden) also modulates intervention outcomes: depressive symptoms diminish physical energy, social motivation, and the willingness to initiate social interaction, while loneliness can worsen depressive symptoms by decreasing perceived social support and amplifying negative cognitive patterns. A meta-analysis confirms a robust reciprocal association between loneliness and depressive symptoms in the general population, supporting the notion that baseline depressive burden may both decrease engagement with GBPA interventions and make the decrease of loneliness harder to sustain without concurrent mood support (Chen et al., 2023). Complementary genetic evidence from Mendelian randomization analyses further determines bidirectional causal links between loneliness and major depression, underscoring that subgroup differences in depressive symptom severity (high vs. low) are likely to be mechanistically meaningful, but potentially significant for GBPA results in terms of mechanisms (Sbarra et al., 2023). Physical function, disability, and health constraints act as additional moderators: functional limitations can reduce the feasibility of GBPA participation, increase fatigue during activities, and increase dependence on instrumental support, possibly altering the significance of the main pathways (as peer support becomes increasingly important when participation obstacles are practical). On the other hand, limited physical abilities can heighten feelings of loneliness by reducing mobility and decreasing chances for daily social engagement. Longitudinal evidence in older adults shows that social isolation and loneliness relate differently to the onset of functional disability (with gender-based variations), implying that baseline functional status and mobility constraints may moderate how GBPA decreases loneliness—whether mainly through increased opportunities for social contact, through fostering in-group identity, or via offering reliable instrumental and emotional peer support (Guo et al., 2021). Finally, baseline loneliness severity and “approach capacity” create a central paradox: the most lonely individuals may have the greatest potential to benefit from GBPA, but also experience the steepest barriers to engagement, driven by social avoidance, mistrust, and low perceived social support. Recent studies in older adults reveal a two-way relationship between physical activity and loneliness, consistent with self-reinforcing cycles (loneliness decreases physical activity, which in turn further increases feelings of loneliness) (Gu and Li, 2025). This motivates the use of a stepped social-dose approach in GBPA design: start with predictable, low-threat social routines to build the belongingness pathway, then gradually introduce identity scaffolding and reciprocal peer support structures as participants’ social approach capacity grows.

#### 3.6.2 Group-level moderators

Group size influences how often members interact and recognize each other. Extremely large groups might lead to anonymity and parallel participation, reducing the sense of support and belonging, while very small groups might enhance intimacy, but also social scrutiny. Research on synchrony indicates that the size of a group can influence the social bonding effects of moving in unison, and meta-analyses on synchrony specifically examine group size as a factor affecting social outcomes (Mogan et al., 2017). While this literature does not specifically address GBPA, it is relevant to the mechanics of activities such as dance and rowing that depend on synchrony. Group-size considerations are also prominent in cohesion research, where structural features can impact perceived cohesion beyond individual differences (Tulin et al., 2018).

Homogeneity may accelerate familiarity and identity formation (“people like me”), while heterogeneity can broaden inclusion and reduce stigma if norms are explicitly inclusive. The main issue is not uniformity itself, but whether the group’s framework enforces a single ‘correct’ way to belong or allows for diverse methods of participation. Social identity theory suggests that the characteristics and boundaries of an identity determine whether a shared identity will be supportive or exclusionary (Schermer et al., 2023).

Research consistently shows that perceptions of group cohesion are connected to sustained participation in exercise and sports, which are essential for building social connections. Cohesion has been shown to predict participation over time in older-adult exercise conceptualizations, and cohesion–adherence associations appear even in comparatively unstructured exercise settings (Estabrooks and Carron, 1999). Reviews of exercise-group cohesion summarize evidence that cohesion relates to behavioral, cognitive, and affective outcomes, reinforcing the view that cohesion is a central group-level moderator instead of a mere correlate (Burke et al., 2008). The motivational climate created by leaders (mastery/task-involving vs. ego/performance-involving) plausibly moderates the development of cohesion and belonging. Meta-analyses in sports show that task/mastery environments are more consistently associated with hedonic well-being than ego environments, reinforcing the idea that mastery climates are more conducive to psychological connection (Lochbaum and Sisneros, 2024). Experimental/field intervention evidence further reveals that coach-initiated climate manipulations can impact athletes’ perceptions of cohesion (Eys et al., 2013). Although these are sport-team data, similar mechanisms are likely relevant in GBPA mastery climates, which reduce evaluative threat and promote psychological safety and inclusive belonging.

Competitive norms can energize identity and joint striving for some groups, but they can also intensify social comparison and exclusion, especially for low-fitness or stigmatized participants. Mixed-studies evidence on loneliness in sport indicates that sport settings can either protect against or contribute to loneliness, depending on their social dynamics, norms, and context (Jackman et al., 2024). This highlights a fundamental moderator principle: the norms of inclusion and psychological safety determine whether social exposure becomes social connection.

#### 3.6.3 Context-level moderators

GBPA is embedded within infrastructure, including transportation, secure venues, affordability, and the presence of culturally suitable groups. Even if a program is theoretically well-designed, limited community resources can constrain the realized social impact (e.g., low retention due to travel difficulties). Implementation-oriented designs (e.g., connecting participants with local choices, adapting to limitations) become part of the intervention’s effective social dose in practice.

Data from different countries suggest that cultural context influences both baseline loneliness levels and the interpretation of social connection. Research based on international survey data implies that individuals from collectivist countries tend to report loneliness less often, and collectivism could also shape family and marital dynamics (Taniguchi and Kaufman, 2022). A multi-country study examining horizontal/vertical collectivism and individualism similarly found small but significant associations with loneliness across 28 countries, with horizontal collectivism generally predicting lower loneliness (Schermer et al., 2023). Research in cultural psychology suggests that both individualism and collectivism may involve risks based on beliefs about ‘how one should be socially integrated,’ indicating that GBPA identity and norm design might require cultural customization (e.g., focusing on the role of collective contribution as opposed to personal advancement) (Heu et al., 2019).

Digital elements can extend interaction beyond in-person meetings and sustain identity continuity, but they may also intensify social comparison and create surveillance-like pressure. Evidence from large-scale behavioral data on Strava reveals that social interactions (e.g., “kudos,” clubmate activity) shape exercise behavior—indicating that online social structures can change engagement and possibly perceived connection (Franken et al., 2023). Studies on online fitness emphasize the ‘sense of community’ as an important factor related to participation in connected fitness brand ecosystems (Davies et al., 2024). The key question is whether online interactions support inclusion, peer support, and identity continuity, or heighten evaluative threat and thereby undermine belongingness.

#### 3.6.4 Activity-type moderators

Different activities create different social structures: cooperative formats foster interdependence and shared goals, whereas selection-based or adversarial formats may amplify hierarchy and exclusion. A systematic review on sports participation and mental health/social outcomes highlights different associations between team and individual sports, supporting the notion that ‘the social architecture of the activity matters (Eather et al., 2023). For loneliness specifically, the review of mixed studies on sport and loneliness emphasizes differences across different settings and underscores mechanisms such as social inclusion/exclusion, identity, and support dynamics (Jackman et al., 2024).

Skill threshold (high vs. low barrier): Activities that require high levels of skill or competence can create feelings of inadequacy and shame for newcomers, weakening their sense of belonging and increasing dropout rates. Activities with low entry barriers, such as walking groups, beginner dance classes, and adapted exercises, might be more effective in fostering initial feelings of belonging and gradual identity development, particularly for participants experiencing high levels of loneliness or anxiety.

Synchronous movement is strongly associated with social bonding and prosocial actions. A meta-analysis of synchrony effects shows that synchronized movement/vocalization increases perceived bonding and prosocial behavior relative to non-synchronous conditions, and examines moderators comprising group size (Mogan et al., 2017). Studies show that synchrony can strengthen social ties across group boundaries, implying that rhythmically synchronized GBPA could provide a greater bonding yield per unit of social exposure (Tunçgenç and Cohen, 2016). This provides a mechanistic rationale for prioritizing synchronous formats when the main objective is to reduce loneliness, provided that inclusive norms are maintained.

Not all competition is harmful: emerging quasi-experimental evidence reveals that structured team-based competitive sports may decrease adolescent loneliness via increased “public belonging,” showing that competition may support identity and belonging under some conditions when norms and facilitation are supportive (Liang et al., 2026). These moderating patterns suggest that a competitive framework needs to be combined with a mastery-oriented atmosphere and inclusion protections to prevent exclusionary behaviors.

### 3.7 Measurement and design implications

#### 3.7.1 Outcome measurement recommendations

Measurement recommendations for GBPA interventions targeting loneliness are summarized below. To facilitate mechanism-sensitive intervention design and evaluation, Table 1 summarizes the major social-dose components, their hypothesized psychosocial pathways, example intervention techniques, and candidate measures.

**Table 1:** Social dose components, hypothesized mechanisms, example intervention techniques, and candidate measures.

Component cluster	Social dose component	Hypothesized mechanism pathway(s)	Example intervention techniques	Candidate measures/process indicators
Structural	Stable cohorts	Belongingness; Social identity; Peer support	Closed-group enrolment; fixed class rosters; stable pods or teams across sessions	Proportion of repeated co-attendance; group-member continuity rate; attendance regularity; participant-reported familiarity with other members
Structural	Repeated co-presence	Belongingness; Peer support	Regular session schedule; minimum attendance blocks; recurring paired or subgroup activities	Session frequency, cumulative shared attendance exposure, co-attendance density, perceived familiarity
Structural	Predictable routines	Belongingness	Standardized opening/closing rituals; consistent session scripts; recurring milestones	Participant ratings of predictability and comfort; routine fidelity checklist; perceived social safety
Relational/process	Psychologically safe climate	Belongingness; Peer support	Instructor uses inclusive language; normalizes varied ability levels; explicitly discourages shaming and ridicule	Psychological safety ratings; perceived inclusiveness; observed leader behaviors; reports of evaluative threat
Relational/process	Low-threat interaction opportunities	Belongingness; Peer support	Brief structured check-ins; guided introductions; pair-share prompts; post-session reflection questions	Frequency of interaction episodes; proportion of participants engaging in interaction; perceived ease of initiating conversation
Symbolic/identity	Shared goals and joint striving	Social identity: Belongingness	Collective step/distance targets; group challenge events; milestone celebrations	Goal commitment, perceived shared purpose, group identification scores, and attendance during milestone periods
Symbolic/identity	Group symbols and rituals	Social identity	Group name; team color/logo; badges; shared phrases; recurring symbolic rituals	Identity salience; perceived group distinctiveness; symbolic participation rate; qualitative reports of “we-ness.”
Symbolic/identity	Role differentiation and valued contribution	Social identity: Belongingness	Peer greeter; pace guide; new-member companion; equipment helper; check-in organizer	Role uptake rate, perceived contribution value, role clarity, and retention among lower-confidence participants
Support/facilitation	Buddy systems	Peer support; Belongingness	Pairing newcomers with returning members for the first 2–4 sessions; mutual check-ins; reminder partnerships	Buddy contact frequency; perceived support availability; newcomer retention; companionship ratings
Support/facilitation	Peer mentors/peer volunteers	Peer support; Belongingness; Social identity	Trained peer volunteers; absence follow-up; supportive outreach; newcomer integration support	Mentor-participant contact logs; perceived peer support; acceptability; adherence and retention
Support/facilitation	Leader-facilitated connection	Belongingness; Peer support; Social identity	Active introductions; encouragement of inclusive norms; gentle interaction scaffolding; early management of exclusionary behavior	Instructor fidelity checklist; participant ratings of leader support; observed inclusive facilitation behaviors
Support/facilitation	Online continuity channels	Social identity; Peer support	Group chat; moderated online community; between-session encouragement; reminders framed as support rather than surveillance	Message engagement, ratio of supportive to comparative content, perceived continuity between sessions, and digital acceptability
Cross-cutting	Normative climate of inclusion	Belongingness; Social identity; Peer support	Explicit anti-shaming rules; inclusive onboarding scripts; mastery-oriented climate; diversity-affirming norms	Inclusion climate scales, reports of exclusion or clique formation, perceived fairness, and dropout reasons
Cross-cutting	Social dose as an integrated exposure	All three pathways	Combining stable structure + interaction scripts + identity scaffolding + support roles in one intervention package	Composite social-dose index; pathway measures collected longitudinally; mediation analyses linking exposure, mechanisms, and loneliness outcomes

For GBPA interventions targeting loneliness, measurement should prioritize conceptual clarity and sensitivity to intervention-related change, starting with the primary outcome of loneliness. This subjective construct can be operationalized as overall loneliness or decomposed into emotional vs. social loneliness, requiring an initial conceptual decision about which aspect of loneliness the intervention’s “social dose” aims to target; for narrative synthesis across heterogeneous studies, we suggest categorizing loneliness measures into three groups and clearly tracking them (i) dimensionality, (ii) item wording (direct vs. indirect), and (iii) reference period. The UCLA Loneliness Scale family is among the most commonly used measures of loneliness in intervention research, with the 20-item UCLA Loneliness Scale Version 3 acting as the standard measure for loneliness in intervention research, backed by robust reliability and validity evidence (Russell, 1996); short forms (e.g., ULS-8) are generally used to decrease participant burden, which can be acceptable, but may alter item content and factor emphasis (Wu and Yao, 2008), whereas the three-item UCLA-based short scale (Hughes et al.) is suitable for large surveys and repeated assessments when space is limited, performing well for overall loneliness, but lacking granularity for mechanism testing (Hughes et al., 2004). The UK Office for National Statistics recommends using the UCLA 3-item set together with a direct loneliness question where feasible, and using only the direct question when only one item can be used, as a practical method for trials and regular program evaluations (Office for National Statistics, 2018). The De Jong Gierveld Loneliness Scale offers an important advantage by explicitly separating emotional and social loneliness, which aligns well with the present framework, as belongingness, identity, and support pathways may influence these dimensions differently (de Jong and van Tilburg, 2008). Direct single-item loneliness measures (“How frequently do you find yourself feeling lonely?”) are progressively used in population tracking and pragmatic settings, but should be seen as a universal self-label that is sensitive to stigma and cultural norms, and is most justifiable when used alongside indirect items (such as UCLA-3) as advised by ONS guidance (Office for National Statistics, 2018). To manage various loneliness scales in a narrative review while preserving comparability without conducting a meta-analysis, we suggest three steps: Step 1: Categorize the loneliness outcome of each study as either overall or emotional/social (if DJGLS is used) and as direct or indirect wording (Russell, 1996); Step 2: Standardize the analysis by indicating the direction of change, assessing if the change is clinically or practically significant as defined by the authors, and determining if the effects last at follow-up (≥3 months); Step 3: Include a sensitivity narrative highlighting that ultra-short measures (1–3 items) have less detail for testing mechanisms and are more prone to wording effects, according to national measurement guidance (Office for National Statistics, 2018). Furthermore, objective social isolation should be assessed together with loneliness to prevent conflating one construct with the other, considering the moderate link between subjective loneliness and objective isolation noted in the development of concise loneliness scales (Hughes et al., 2004), with the LSNS-6 (Lubben Social Network Scale-6) serving as a pragmatic, widely used isolation screener that quantifies family/friend network size and contact and has been cross-culturally validated (Lubben et al., 2006). To evaluate the logic of ‘exercise dose→social dose→pathways→loneliness,’ it is important to directly measure mechanistic indicators for the three pathways: for peer support/perceived support availability, the MOS Social Support Survey offers functional subscales (emotional/informational, tangible, affectionate, effective social interaction) and an overall index useful for mapping support categories to mechanisms (Sherbourne and Stewart, 1991), the MSPSS is a concise tool that distinguishes perceived support from family, friends, and significant others, which is beneficial when peer support is expected to be central (Wilcox, 2010). The General Belongingness Scale (GBS) includes facets of acceptance and rejection that align well with the belongingness mechanisms in GBPA (MacKinnon, 2012). SDT-based relatedness or need-satisfaction scales can serve as supplementary indicators of perceived connection (Zajonc, 1968). To examine mechanisms of social identity in exercise groups, validated social identification measures should be used (Dahlem et al., 1991). A pragmatic, minimum-mechanistic assessment set for most GBPA trials would include loneliness as the primary outcome; LSNS-6 for social isolation; one support measure; one belongingness or relatedness measure; and one group identification measure (Russell, 1996).

#### 3.7.2 Design recommendations for mechanism-sensitive GBPA research

**Figure 3:**
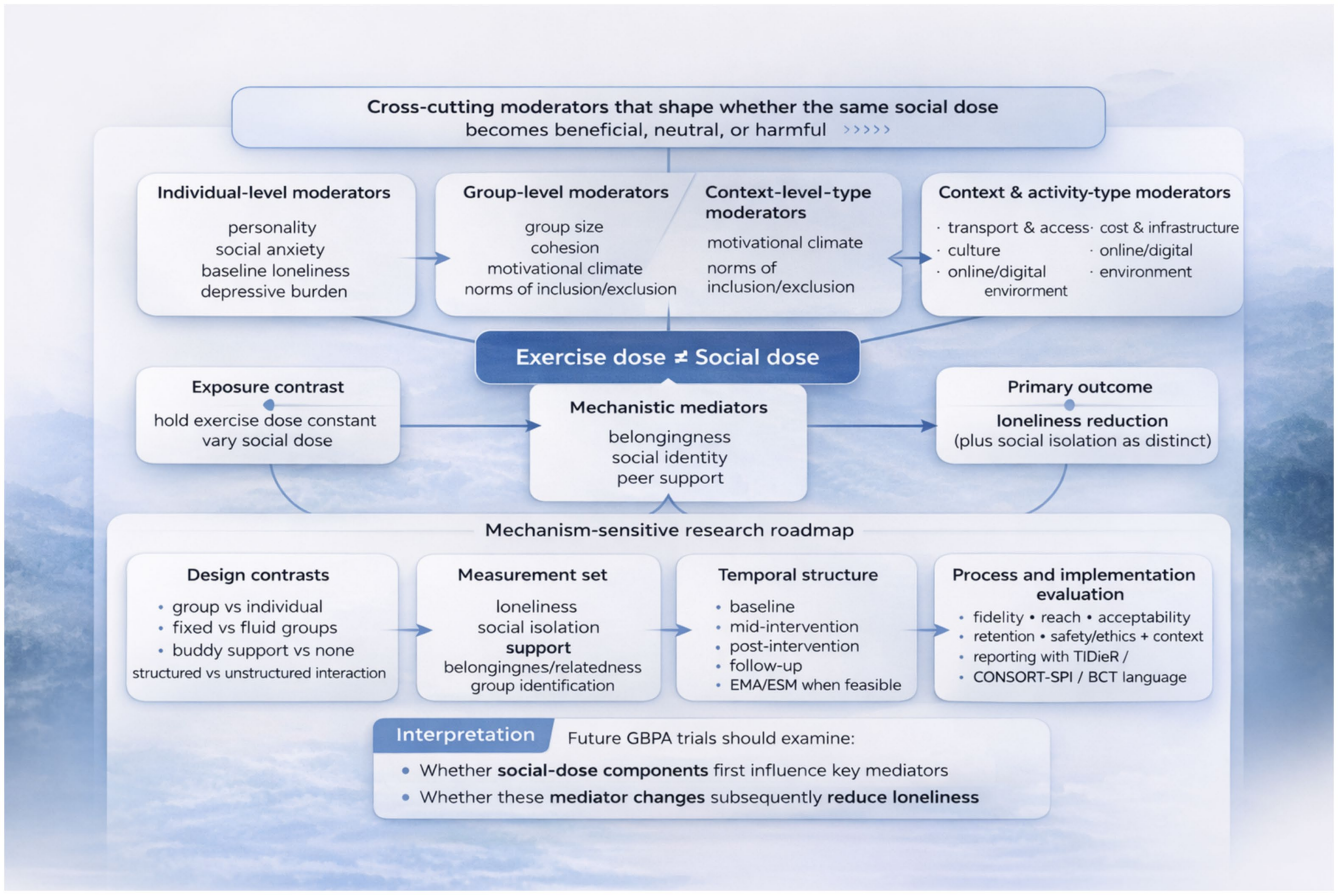
Cross-cutting moderators and a mechanism-sensitive research roadmap for testing social dose in group-based physical activity interventions targeting loneliness.

To test the roles of belongingness, identity, and support in explaining change in loneliness more rigorously, longitudinal mediation designs that establish clear temporal ordering are preferable to cross-sectional mediation approaches, as classic guidance cautions against the limitations of cross-sectional mediation and advocates for multi-wave models with careful specification (Cole and Maxwell, 2003). In practice, this involves conducting at least three waves of data collection (baseline, during the intervention, and after the intervention, with a follow-up if possible) to examine the key assumption that changes in pathways precede changes in loneliness (Cole and Maxwell, 2003) and using structural equation modeling (SEM) or longitudinal mediation frameworks that follow established mediation-analysis principles (MacKinnon, 2012). Complementing this, intensive longitudinal approaches, such as ecological momentary assessment, may be useful for capturing momentary social dose in real time, as loneliness and social connection can vary across contexts, and retrospective reports are vulnerable to recall bias. Conversely, EMA provides real-time, naturalistic sampling that is well-suited to examining the within-person relationship between daily activity involvement and perceived connection and loneliness in GBPA. This can be operationalized by assessing momentary loneliness, perceived availability of support, belonging, and the prominence of identification on days with sessions compared to days without sessions, and by linking these actions to attendance and exposure during session interactions. Because GBPA is a complex intervention with multiple interacting components and strong contextual dependence, mixed methods approaches paired with process evaluation are important to clarify intervention fidelity, reach, pathways, and conceptualization constraints, with the MRC guidance offering a structured conceptualization and practical recommendations for conducting process evaluation in such complex interventions (Moore et al., 2015). To enhance the testability of the analytical framework, future studies should distinguish more clearly between social dose and exercise dose by using explicit design contrasts: comparing group and individual exercise formats while holding exercise dose constant and varying social exposure, focusing on scenarios with matched exercise frequency, intensity, and duration but different levels of social exposure (e.g., fixed groups with a partner system compared to open sessions without specified interaction), and using factorial or stepped designs to sequentially introduce social-dose components (e.g., beginning with consistent groups, then incorporating identity markers, and finally introducing structured peer support). Finally, rigorous reporting standards are essential for improving reproducibility, particularly because GBPA studies often underreport social and interactional components, particularly for the “social dose” component, as GBPA papers generally underreport social and interactional elements; this involves using the TIDieR checklist to ensure comprehensive and replicable intervention description (Hoffmann et al., 2014), the BCT Taxonomy v1 to define techniques for behavior change and support (e.g., altering the social environment and offering social support) in a standardized language (Michie et al., 2013), and the CONSORT-SPI 2018 extension should be used for trials involving social or psychological interventions (CONSORT cluster extensions are employed if cluster designs are used, a frequent practice in community classes) (Montgomery et al., 2018). Figure 3 shows Cross-cutting moderators and a mechanism-sensitive research roadmap for testing social dose in GBPA interventions targeting loneliness.

## 4 Conclusion and outlook

Group-based physical activity may reduce loneliness not simply by increasing exercise, but by providing a social component that can be deliberately designed, strengthened, or weakened. The core argument of this review is that when the target outcome is loneliness, focusing solely on exercise prescription parameters such as frequency, intensity, and duration is insufficient to explain variation in effectiveness. A key determinant of loneliness reduction may be whether the group setting transforms repeated interaction into sustained connection. We conceptualize this process as beginning with the structural features of GBPA, operating through proximal social processes, and unfolding through three interwoven pathways: belongingness, social identity, and peer support. The social identity pathway focuses on the feeling of being part of a significant ‘we’, and the peer support route includes receiving need-matched support and perceiving it as available. Within this framework, two participants may both join the group exercise. Yet, one develops a genuine connection while the other remains lonely in the group—a difference generally driven by whether the social dose is appropriately designed and aligned with participants’ vulnerabilities and conceptualization constraints.

For practitioners, the main takeaway is to design and manage group physical activity programs as social-connection interventions rather than merely adding informal social elements to exercise sessions. A sense of belonging comes from environments that are stable, low-threat, and predictable; having fixed group structures, consistent opening and closing rituals, structured onboarding scripts for newcomers, and clear, inclusive norms, which is likely to be more beneficial than having ‘more free social time. Social identity is formed through structures of meaning and identity; shared objectives, group symbols, role assignments, and collective milestones transform participation from merely ‘attending a class’ to ‘engaging in a shared activity,’ thereby strengthening the durability of connection. The effectiveness of peer support may depend in part on its availability and responsiveness; structured dyadic partnerships, reciprocal feedback, and facilitators’ connection-promoting behaviors (active introductions, gently building interaction opportunities, timely addressing exclusion and shaming comparisons) are critical. Notably, the quality of support generally outweighs quantity: when interactions lack responsiveness, support does not match participants’ needs, or the climate discourages vulnerability, frequent social contact fails to reduce loneliness and may instead intensify feelings of being misunderstood.

For researchers, future work should move beyond repeatedly asking whether group exercise may work, and instead treat social dose as an intervention component as important as exercise dose, to be measured, reported, and mechanistically tested. Research should include mechanistic indicators along with loneliness outcomes at the measurement level to determine not just whether change occurs, but also through which pathways. This requires comprising social support, belongingness, and social identity as process variables, and characterizing social dose using metrics of group environment and interaction quality, such as group stability, interaction density, relationship quality, normative climate, and identity continuity. At the design level, inferential rigor can be improved using comparison structures that differentiate exercise dose from social dose: for example, comparing group versus individual formats while matching exercise dose, or systematically varying social dose components within the same exercise framework (e.g. fixed groups compared to fluid groups, dyadic support versus lack of defined support, structured interaction scripts versus unstructured ones, identity scaffolding versus absence of shared identity). Longitudinal mediation models should assess whether changes in mechanisms occur before changes in loneliness. Methodologically, intensive longitudinal designs and mixed methods offer unique value: loneliness and connectedness vary across situations, and core mechanisms operate in moment-to-moment interactions rather than in retrospective accounts. Qualitative evidence can provide insights into why certain elements do not work in particular groups or cultural settings, helping to transform the abstract idea of ‘social dose’ into practical design specifics.

Looking forward, one promising future research agenda is to develop social dose into a quantifiable, comparable, and transferable design language, and to explore thresholds and combinations of its minimum effective social dose. The social needs and tolerance for social exposure differ among groups: people with social anxiety or high sensitivity to shame might gain more from interactions that are low-threat and well-structured; individuals with chronic conditions or functional limitations might depend more on practical assistance and ongoing peer companionship; teenagers are more susceptible to being excluded by peers and comparing themselves online, necessitating clearer norm governance and protective measures. Future evidence generation should therefore prioritize equity and accessibility, systematically evaluating which social dose designs inadvertently raise barriers or produce selective retention, which leaves individuals most in need of reducing loneliness less able to benefit from it. Ethics and risk tracking should also become standard: as programs enhance peer connections, add online components, or promote deeper self-disclosure, there may be increased risks of blurred boundaries, overreliance, privacy violations, and exclusionary group dynamics. Only by advancing in parallel across four pillars—effectiveness, pathways, implementation, and safety—can GBPA interventions realize their potential as scalable public health solutions to address loneliness as a significant public health challenge.

Overall, GBPA is most likely to reduce loneliness when exercise is paired with a deliberately designed social environment that fosters familiarity, reciprocity, shared meaning, and perceived support.
